# The thermodynamics of Pr55^Gag^-RNA interaction regulate the assembly of HIV

**DOI:** 10.1371/journal.ppat.1006221

**Published:** 2017-02-21

**Authors:** Hanumant S. Tanwar, Keith K. Khoo, Megan Garvey, Lynne Waddington, Andrew Leis, Marcel Hijnen, Tony Velkov, Geoff J. Dumsday, William J. McKinstry, Johnson Mak

**Affiliations:** 1 School of Medicine, Deakin University, Geelong, Australia; 2 CSIRO Manufacturing, Parkville, Victoria, Australia; 3 CSIRO Australian Animal Health Laboratory, Geelong, Australia; 4 GE Health, Life Science, Burnley, Australia; 5 Monash Institute of Pharmaceutical Science, Parkville, Victoria, Australia; 6 CSIRO Manufacturing, Clayton, Victoria, Australia; Vanderbilt University School of Medicine, UNITED STATES

## Abstract

The interactions that occur during HIV Pr55^Gag^ oligomerization and genomic RNA packaging are essential elements that facilitate HIV assembly. However, mechanistic details of these interactions are not clearly defined. Here, we overcome previous limitations in producing large quantities of full-length recombinant Pr55^Gag^ that is required for isothermal titration calorimetry (ITC) studies, and we have revealed the thermodynamic properties of HIV assembly for the first time. Thermodynamic analysis showed that the binding between RNA and HIV Pr55^Gag^ is an energetically favourable reaction (ΔG<0) that is further enhanced by the oligomerization of Pr55^Gag^. The change in enthalpy (ΔH) widens sequentially from: (1) Pr55^Gag^-Psi RNA binding during HIV genome selection; to (2) Pr55^Gag^-Guanosine Uridine (GU)-containing RNA binding in cytoplasm/plasma membrane; and then to (3) Pr55^Gag^-Adenosine(A)-containing RNA binding in immature HIV. These data imply the stepwise increments of heat being released during HIV biogenesis may help to facilitate the process of viral assembly. By mimicking the interactions between A-containing RNA and oligomeric Pr55^Gag^ in immature HIV, it was noted that a p6 domain truncated Pr50^Gag Δp6^ is less efficient than full-length Pr55^Gag^ in this thermodynamic process. These data suggest a potential unknown role of p6 in Pr55^Gag^-Pr55^Gag^ oligomerization and/or Pr55^Gag^-RNA interaction during HIV assembly. Our data provide direct evidence on how nucleic acid sequences and the oligomeric state of Pr55^Gag^ regulate HIV assembly.

## Author summary

Formation of any virus particle will require energy, yet the precise biophysical properties that drive the formation of HIV particles remain undefined. Isothermal titration calorimetry (ITC) is a biophysical technique that is the gold standard to reveal parameters governing biochemical and biophysical reaction. However, ITC requires large amount of proteins for analysis. As large quantities of full-length recombinant HIV Pr55^Gag^ proteins have not been available in the past 30 years due to technical limitation, a comprehensive thermodynamic analysis of full-length HIV Pr55^Gag^ has not been possible. Here, we have generated sufficient amount of full-length recombinant HIV Pr55^Gag^ protein for isothermal titration calorimetry analysis. Our analyses have shown that the major interactions amongst HIV proteins and RNA sequences during viral assembly are energetically favourable reactions. In other words, HIV Pr55^Gag^ proteins and viral RNA have evolved to overcome the energy barrier for virus formation by utilising energy obtained from protein-RNA interactions in order to facilitate the viral assembly process. Furthermore, HIV also use the oligomeric states of HIV Pr55^Gag^ proteins and the RNA sequences as means to regulate the viral assembly process.

## Introduction

The assembly of the HIV particle is orchestrated by the HIV-1 Gag precursor protein (Pr55^Gag^). As the major structural protein forming the virus, it is essential that Pr55^Gag^ packs tightly with other Pr55^Gag^ molecules to shape the virus shell while also encapsulating the genomic RNA required for its replication. Fittingly, Pr55^Gag^ is comprised of multi-functional domains that facilitate key interactions involved in the early stages of the assembly process. The nucleocapsid (NC) domain is responsible for genomic RNA packaging and the capsid (CA) domain mediates the key interactions to promote Pr55^Gag^-Pr55^Gag^ oligomerization (for review see [1–3]). While both Pr55^Gag^-nucleic acid and Pr55^Gag^-Pr55^Gag^ interactions have been identified as major contributors to the assembly process [4], the mechanistic details of how they regulate HIV assembly are not well defined, specifically in the context of full length Pr55^Gag^.

It is generally accepted that nucleic acid binds to Pr55^Gag^ and acts as a scaffold for Pr55^Gag^ oligomerization [5–7], although short oligonucleotides (10 nucleotides or less) are not sufficient to bridge multiple Pr55^Gag^ molecules to support assembly [8, 9]. The p6 C-terminus truncated recombinant Gag protein (Pr50^GagΔp6^), with the addition of nucleic acid and lipid-mimicking inositol phosphate, has previously been shown to assemble *in vitro*, forming virus-like particles (VLPs) in the absence of other viral proteins [7, 8]. Cellular imaging and immunoprecipitation studies with virion producing cells have suggested that nucleic acid initially binds to cytosolic Pr55^Gag^, therefore promoting the formation of low order oligomers of Pr55^Gag^ in the cytosol; higher order Pr55^Gag^ oligomers only occur upon Pr55^Gag^ binding to the plasma membrane [10–13]. However, cytoplasmic HIV-1 RNA has also been shown to traffic to the membrane by passive diffusion independent of Pr55^Gag^, suggesting that the viral RNA genome may bind to Pr55^Gag^ molecules on the plasma membrane where a high local concentration of Pr55^Gag^ has already been achieved for oligomerization [14]. At the plasma membrane, the assembly of Pr55^Gag^ has also led to the reorganization of the lipid membrane, such as the nano-clustering of phosphatidyl inositol (4,5) biphosphate lipid [15]. Clearly, the relationships between nucleic acid binding and Pr55^Gag^ oligomerization that lead to virus assembly are still unclear. Moreover, the energy requirements and thermodynamic properties that drive these two basic components to facilitate the formation of the viral particle are currently not known. In this regard, a thermodynamic analysis of the assembly process will provide critical information (such as, the affinity and the stoichiometry between ligands and substrates, plus the energetics involved in these reactions) to define the mechanisms of the process. Specifically, enthalpic and entropic components of the binding reaction derived from the analysis will enable us to gain insight into the mechanisms of the interactions that take place.

Apart from the packaging signal (Psi) of the genomic RNA that is involved in the encapsidation of viral genome, the contributions from the rest of the viral RNA sequence to the assembly process is not well defined. Early studies have indicated that the mature nucleocapsid domain on its own displays preferential binding to selected nucleic acid sequence motifs in addition to the Psi packaging sequences [9, 16, 17]. Studies comparing Psi and non-Psi RNA binding to C-terminal truncated Pr55^Gag^ protein reported differences in the electrostatic and non-electrostatic interactions of Pr55^Gag^ proteins with different RNAs [18], and we have shown how Pr55^Gag^ selected between unspliced and spliced HIV RNA *in vitro* for viral assembly [19, 20]. Recent cross-linking immunoprecipitation experiments have shown that different RNA motifs interact with Pr55^Gag^ at different stages of virus biogenesis [21]. However, the functional significance and the mechanisms of these specific Pr55^Gag^-RNA interactions in regulating the assembly of Pr55^Gag^ have yet to be shown.

A key bottleneck in acquiring fundamental information about the mechanistic details of Pr55^Gag^ assembly has been the limited availability of full-length recombinant Gag protein for analysis, as large quantities of full-length recombinant Pr55^Gag^ are difficult to produce [8, 22]. Here, we have produced large quantities of recombinant Pr55^Gag^ and use *in vitro* systems to study the early steps of HIV assembly. Using the entire form of recombinant Pr55^Gag^ in isothermal titration calorimetry (ITC) studies, we present the first thermodynamic analysis of how HIV Pr55^Gag^ and RNA regulate assembly. More specifically, we directly demonstrate that: (1) the specific interaction between Pr55^Gag^ and Adenosine (A)-containing RNA motifs; and (2) the oligomerization of Pr55^Gag^; are energetically favourable reactions that facilitate virion particle formation. Our findings benchmark the thermodynamic regulations of retroviral assembly, and provide a platform to interrogate, and ultimately to reveal, structural and biophysical properties between HIV Pr55^Gag^ and host cell factors during viral replication.

## Results

### Pr55^Gag^-RNA binding and Pr55^Gag^-oligomerization during HIV assembly is an energetically favourable process

The binding of RNA to Pr55^Gag^ and the oligomerization of Pr55^Gag^ molecules are two of the basic components of viral assembly. However, a thermodynamic analysis unpacking how these two components drive the assembly process has never been conducted in the context of full length Pr55^Gag^. We produced sufficient quantities of recombinant full-length Pr55^Gag^ (**S1 Fig**) and validated its capacity to oligomerize and form VLPs in the presence of RNA *in vitro*, similar to previous observations with the truncated Pr50^GagΔp6^ [8, 23–26](**S2 Fig**).

**Fig 1:**
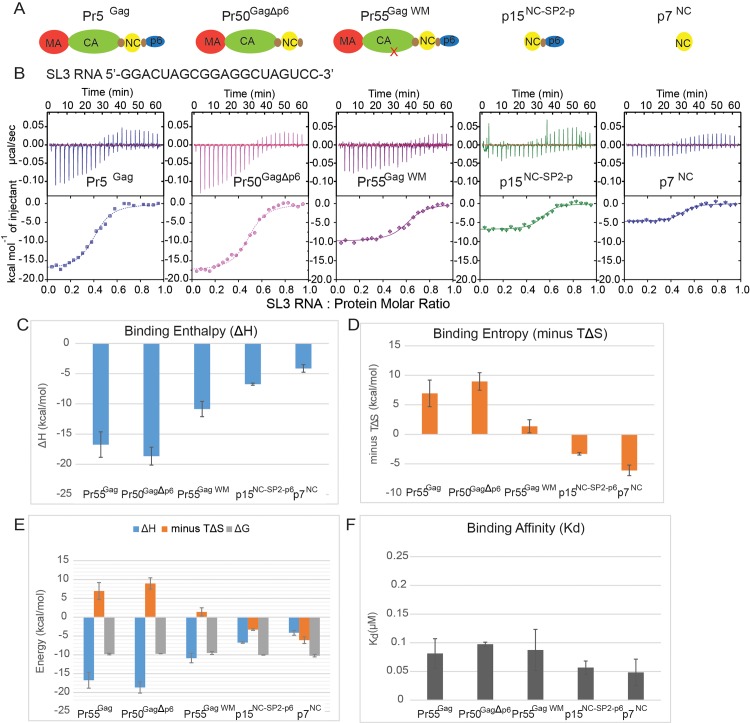
Binding between Psi SL3 RNA and Pr55^Gag^ is an energetically favourable reaction and the level of energy released is enhanced by the oligomerization capacity of Pr55^Gag^. **(A)** Schematic representation of domain arrangement in Gag proteins (Pr55^Gag^, Pr50^GagΔp6^, Pr55^Gag WM^) and processed NC proteins (p15^NC-SP2-p6^ and p7^NC^) used in this study **(B)** The top panels show the differential heat released following baseline subtraction and the bottom panels show representative ITC binding curve indicating heat released per mole of oligonucleotide titrated when Psi SL3 RNA (40 μM) was injected in 1.5 μL aliquots into 8 μM of Pr55^Gag^, Pr50^GagΔp6^, Pr55^Gag WM^, p15^NC-SP2-p6^ or p7^NC^, respectively. Calculated **(C)** binding enthalpies (ΔH), **(D)** binding entropies (minus TΔS), **(E)** Gibb’s free energies (ΔG) and **(F)** binding affinities (*k*_d_) from ITC binding curves fitted to a one-site interaction model plotted for replicate Gag protein interactions with Psi SL3 RNA (n = 3). The mean value is plotted and the error bars represent the SEM.

Using ITC, we first characterized the binding between Pr55^Gag^ protein (domain arrangements of Pr55^Gag^ proteins used schematized in **Fig 1A**) and the packaging signal stem loop 3 RNA (Psi SL3 RNA), a region of the viral RNA involved in genomic packaging and known to bind to NC with high affinity [17, 27]. Representative ITC curves were shown in **Fig 1B**, while data in **Fig 1C–1F** reflect the means of all three experiments. The representative ITC curves in **Fig 1B** are the raw values prior to adjustments (such as fluctuations of detected temperature of the reaction; fluctuation of cabinet temperature of the ITC chamber for each point of the measurement; and the normalization of baseline temperature with the corresponding buffer controls). The ITC curves also acted as quality assurance with these ITC experiments, demonstrating the reliability of the data. We first benchmarked the thermodynamics properties of HIV assembly using Pr55^Gag^ molecules that are capable of oligomerizing (Pr55^Gag^ and Pr50^GagΔp6^) and Psi SL3 RNA that is the primary determinant for genomic RNA selection in the cytoplasm. Both Pr55^Gag^ and Pr55^GagΔp6^ displayed a favourable binding enthalpy [ΔH ~ -17 kcal mol^-1^] as measured by the maximum heat energy release during ITC titrations of Psi SL3 RNA into the Pr55^Gag^ protein (**Fig 1B and 1C**). The reaction is also thermodynamically favourable with ΔG<0. The detected energy release in these reactions is likely to be the sum of both Pr55^Gag^-RNA interaction and Pr55^Gag^-Pr55^Gag^ oligomerization. To estimate the fraction of energy released that is contributed by Pr55^Gag^-RNA vs Pr55^Gag^-Pr55^Gag^ oligomerization, we have used a mutant, Pr55^Gag WM316-7AA^ (or Pr55^Gag WM^) [28] that is incapable of supporting the dimerization of Pr55^Gag^ via the CA-hexamer dimerization domain. Therefore, mutant Pr55^Gag WM^ is known to partially suppress the assembly of HIV via the dimerization defect of the CA hexamers [4]. As measured by the maximum heat energy release during ITC titrations of Psi SL3 RNA into the Gag protein, interaction of Psi SL3 RNA with Pr55^Gag WM^ displayed a favourable binding enthalpy [ΔH ~ -10 kcal mol^-1^] (**Fig 1B and 1C**), but it was only 60% of the energy release of the wild type Pr55^Gag^-RNA interaction (**Fig 1B and 1C**). These data implied that the oligomerization of Pr55^Gag^ during Pr55^Gag^-RNA interaction contributed to at least 40% of energy detected in the ITC experiment, and the Pr55^Gag^- Pr55^Gag^ interactions increased its favourable binding enthalpy with Psi SL3 RNA. In support of this, interaction of Psi SL3 RNA with both processed NC proteins (p15^NC-SP2-p6^ and p7^NC^ that lack the CA-CA interaction domains for oligomerization) also displayed less favourable binding enthalpies [ΔH ~ -5-6 kcal mol^-1^] compared to the full-length Gag proteins Pr55^Gag^.

The interaction of Pr55^Gag^ and Pr50^Δp6Gag^ with Psi SL3 RNA however had a more unfavourable entropic contribution compared to that of Pr55^Gag WM^ (**Fig 1D**). As entropy is a measure of the disorder of the reaction [29], the unfavourable entropy likely results from loss of conformational freedom due to Pr55^Gag^-Pr55^Gag^ oligomerization. Nevertheless, the larger accompanying favourable enthalpic contributions observed with Psi SL3 binding to Pr55^Gag^ and Pr50^Δp6Gag^ compensates for the unfavourable entropic component to produce an overall favourable Gibbs free energy (ΔG<0) of binding (**Fig 1E**), highlighting that the Pr55^Gag^-RNA (SL3) interaction is an energetically favourable process overall. On the other hand, reactions with processed NC proteins (p15^NC-SP2-p6^ and p7^NC^) were characterized by favourable entropic contributions, likely driven by displacement of ordered water molecules [30] as the result of NC—Psi RNA interaction (**Fig 1D**). The favourable entropic component of Psi SL3 binding to p15^NC-SP2-p6^ and p7^NC^, combined with the accompanying favourable enthalpic component contributed to an overall favourable Gibbs free energy (ΔG<0) of binding.

Interestingly, calculated binding affinities for Pr55^Gag^ [*K*_*d*_ = 0.081 ± 0.025 μM], Pr50^Δp6Gag^ [*K*_*d*_ = 0.098 ± 0.003 μM], Pr55^Gag WM316-7AA^ [*K*_*d*_ = 0.088 ± 0.035 μM], p15^NC-SP2-p6^ [*K*_*d*_ = 0.057 ± 0.011 μM] and p7^NC^ [*K*_*d*_ = 0.048 ± 0.023μM] did not differ significantly (**Fig 1F**). These data imply that the binding between Pr55^Gag^ and SL3 RNA for genomic RNA selection during viral assembly is independent of the oligomerization state of Pr55^Gag^. Our ITC results demonstrate that the binding of Psi SL3 RNA to Pr55^Gag^ is energetically favourable (ΔG<0). Bindings of Psi SL3 to p15^NC-SP2—p6^ and p7^NC^ are results of both favourable entropic and enthalpic contributions, suggesting the involvement of both polar and hydrophobic interactions [30]. By definition, these interactions must occur between the Psi SL3 RNA and the nucleocapsid protein. Reactions of Psi SL3 with full length Pr55^Gag^ that has an increased capacity to oligomerize were driven by a larger favourable enthalpy, which helps in overcoming the unfavourable entropy brought about by conformational restrictions due to oligomerization.

### Pr55^Gag^ displays increased binding preference toward identified Pr55^Gag^-interacting RNA sequence motifs in immature virion over those in cytosol

**Fig 2:**
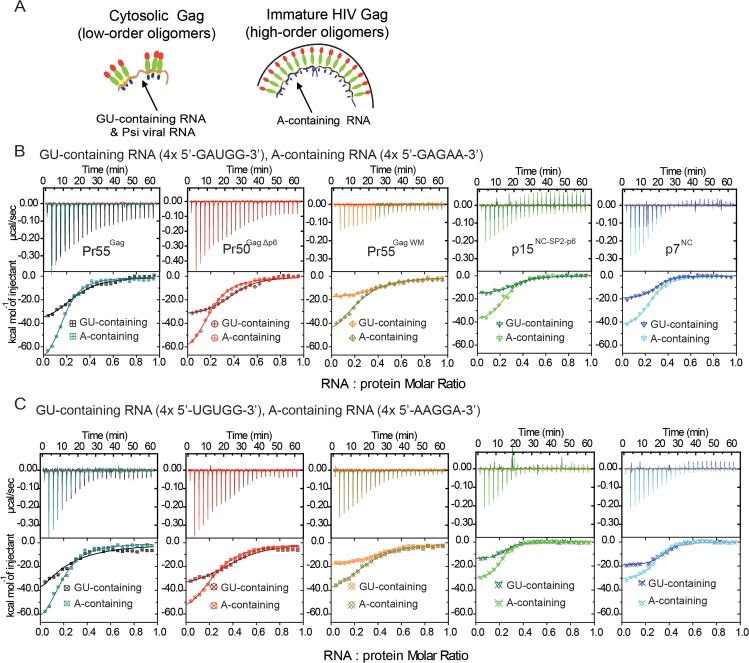
ITC analysis of Pr55^Gag^-RNA binding show more energetically favourable binding of Pr55^Gag^ to A-containing over GU-containing RNA. **(A)** Schematic of Pr55^Gag^-RNA binding specificity during viral assembly. Representative ITC binding curves indicating heat released per mole of oligonucleotide titrated when 40 μM of 20mers; **(B)** 4x 5’-GAUGG-3’ (GU-containing) and 4x 5’-GAGAA-3’ (A-containing) RNA (n = 3);.and **(C)** 4x 5’-UGUGG-3’ (GU-containing) and 4x 5’-AAGGA-3’ (A-containing) RNA were injected in 1.5 ul aliquots into 8 μM of Pr55^Gag^, Pr50^GagΔp6^, Pr55^Gag WM^, p15^NC-SP2-p6^ and p7^NC^, respectively (n = 2).

During the assembly of HIV, there are only 2 sets of SL3 sequences in the packaged HIV dimeric RNA genome. As a result, most of the 1500–2500 Pr55^Gag^ molecules in the assembled virion must bind to other segments of the RNA genome. HIV Pr55^Gag^ has been previously reported to transiently interact with different RNA sequence motifs during the viral assembly process [21]. It has been suggested that cytosolic Pr55^Gag^ consisting of low-order Pr55^Gag^ oligomers interact with Guanosine Uridine (GU)-containing RNA sequence motifs, while high-order Pr55^Gag^ oligomers in the immature virion are more likely to associate with Adenosine (A)-containing RNA sequence motifs (schematized in **Fig 2A**). However, the role these non-packaging segments of the RNA genome play in viral assembly still remains unanswered. These GU-containing RNA sequences and A-containing RNA sequences are also interspersed throughout the HIV genome and in many cellular RNA sequences (**S3 Fig**).

**Fig 3:**
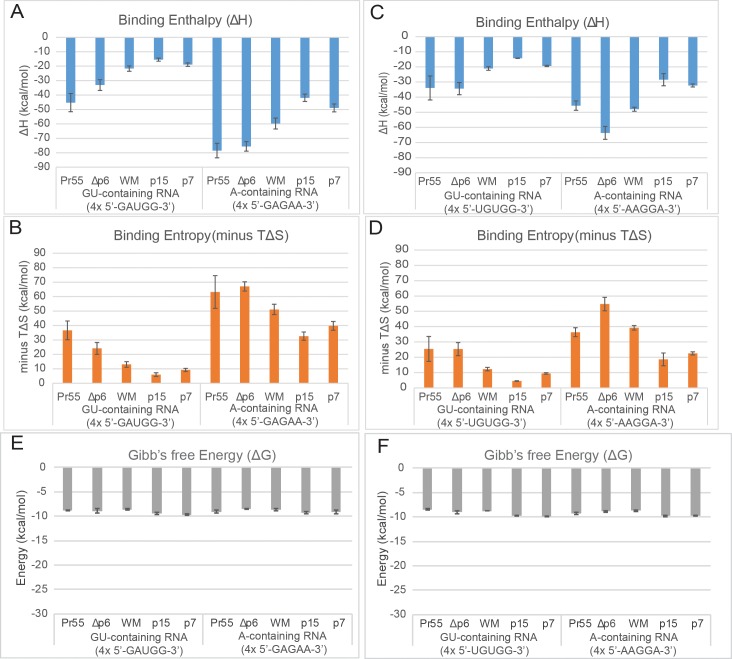
ITC analysis of Gag-RNA binding show more energetically favourable binding enthalpies and larger unfavourable entropy of Gag to A-containing over GU-containing RNA. Calculated **(A)** binding enthalpy, **(B)** binding entropy and **(E)** Gibb’s Free Energy from ITC binding curves fitted to a one-site interaction model plotted for replicate Gag protein interactions with first set of 20mers GU-containing RNA (4x 5’-GAUGG-3’) and A-containing RNA (4x 5’-GAGAA-3’) (n = 3). Calculated **(C)** binding enthalpy and **(D)** binding entropy and **(F)** Gibb’s Free Energy from ITC binding curves with second set of 20mers GU-containing (4x 5’-UGUGG-3’) and A-containing RNA (4x 5’-AAGGA-3’) (n = 2). The mean value is plotted and the error bars represent the SEM.

To investigate whether the thermodynamic relationship with Pr55^Gag^ can provide insight into the function of these sequences, we conducted ITC analyses of Gag interaction with the top 4 RNA sequence motifs that were previously identified to interact with HIV Gag either in the cytosol (GU-containing RNA motifs: 5’GAUGG3’ and 5’UGUGG3’) or within the immature virion (A-containing RNA motifs: 5’GAGAA3’ and 5’AAGGA3’) [21]. Binding of the 20 mer of the GU-containing RNA motif 4x 5’-GAUGG-3’ RNA to the oligomerization-impaired Pr55^Gag WM^ (mimicking the low-order oligomeric form of cytosolic Gag) resulted in an enthalpy release [ΔH ~-20 kcal mol^-1^] (**Figs 2B and 3A**), which is comparable to that released in the binding to processed NC proteins (p15^NC-SP2-p6^ and p7^NC^) (**Figs 2B and 3A**). In contrast, higher enthalpy was released in the binding of the same 20mer GU-containing RNA motif 4x 5’-GAUGG-3’ RNA with the oligomeric forms of Gag (Pr55^Gag^ and Pr50^GagΔp6^) with means of ΔH ~-45 kcal mol^-1^ and -35 kcal mol^-1^, respectively (**Fig 3A**) (or raw values of ΔH ~-35 kcal mol^-1^ and -30 kcal mol^-1^, respectively, in the un-adjusted representative ITC curves, **Fig 2B**), suggesting that oligomerization contributes at least in part to the favourable enthalpy release of Pr55^Gag^ binding with GU-containing RNA. These data are consistent with those observed with Psi SL3 RNA based Pr55^Gag^-RNA ITC studies (**Fig 1**). In support of this, more enthlapy release was also observed in the interaction between the 20 mer of A-containing RNA motif 4x 5’-GAGAA-3’ RNA and the oligomeric forms of Gag (Pr55^Gag^ and Pr50^GagΔp6^), with means of ΔH ~-80 kcal mol^-1^ (**Fig 3A**) (or raw values of ΔH ~-70 kcal mol^-1^ and -60 kcal mol^-1^, respectively, in the un-adjusted representative ITC curves, **Fig 2B**), over the interaction between the 20 mer of A-containing RNA motif 4x 5’-GAGAA-3’ RNA and the oligomeric-deficient forms of Gag (Pr55^Gag WM^)[ΔH ~ -60 kcal mol^-1^] (**Fig 3A**) (or raw value of -40 kcal mol^-1^ in the un-adjusted representative ITC curve, **Fig 2B**). Agreeably, ITC analyses repeated with the second set of GU-containing and A-containing RNA sequence motifs (4x 5’-UGUGG-3’ and 4x 5’-AAGGA-3’, respectively) produced a similar trend in binding enthalpies measured across the Gag constructs (**Figs 2C and 3C**). Our results showed that the favourable enthalpy and Gibbs Free energy release from oligomerization can potentially be a general feature of RNA-Pr55^Gag^ protein interaction during viral assembly.

**Fig 4:**
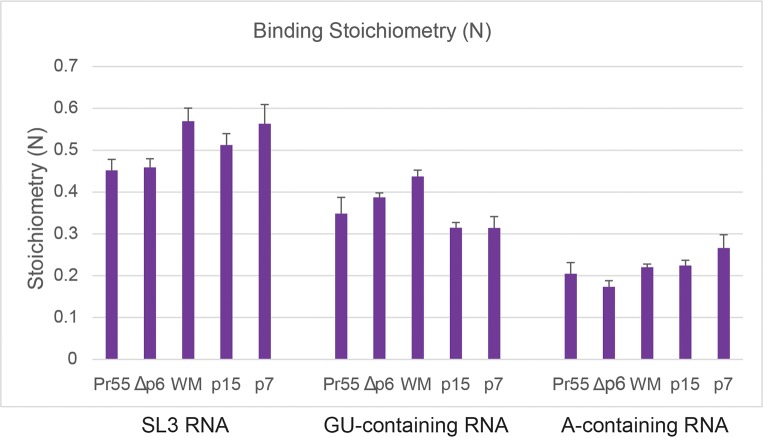
Stoichiometry of binding indicates higher number of Gag binds to A-containing RNA over GU-containing RNA and SL3. Calculated binding stoichiometry (N) from ITC binding curves fitted to a one-site interaction model plotted for replicate Gag protein interactions with SL3 RNA, GU-containing RNA (4’x 5’-GAUGG-3’) and A-containing RNA (4x 5’-GAGAA-3’) (n = 3). The binding stoichiometry (N) refers to RNA:Gag ratio.

Conversely, across the Gag constructs tested independent of their size and oligomeric capacity, thermodynamic analysis also indicated that the interaction of the A-containing RNA motifs with Gag were characterized by about 2-times more favourable enthalpy compared to that of the GU-containing RNA motifs (**Fig 3A and 3C**). This result suggests that the type of RNA sequence interacting with the Gag protein also contributes to the favourable release of enthalpy. Additionally, the type of RNA sequence may also be a determinant of how tightly packed Gag can bind. An analysis of the stoichiometry of binding showed a trend that 1 A-containing RNA molecule would bind to 5 Gag molecules (RNA:Gag N~0.2) in comparison to 1 GU-containing RNA molecule would bind to 3 Gag molecules (RNA:Gag N~0.33) (**Fig 4**). However, it is important to acknowledge that the binding stoichiometry data are related to a single type of GU-containing RNA motif and a single type of A-containing RNA motif, and these motifs are artificially presented in 4 consecutive repeats within a synthetic RNA. The potential importance of different RNA-Gag binding stoichiometry must be validated using multiple different natural A-containing RNA motifs and natural GU-containing RNA motifs within the context of HIV RNA genome.

Furthermore, a larger unfavourable entropy was detected when A-containing RNA motifs were used in ITC experiments compared to GU-containing RNA motifs (**Fig 3B and 3D**), suggesting that these designated A-containing RNA motifs might in part better support tight packing of Pr55^Gag^-RNA and/or Pr55^Gag^-Pr55^Gag^ interaction during the biological process, leading to greater loss of conformational freedom. Nevertheless, the larger favourable binding enthalpy associated with binding of A-containing RNA motifs to Pr55^Gag^ overcomes the unfavourable entropy to drive the reaction forward. These differential thermodynamic relationships between Pr55^Gag^ and A-containing RNA motifs in immature virus vs Pr55^Gag^ and GU-containing RNA motifs in cytoplasm have provided insight on how viral particle formations are regulated. The Gibb’s free energy (ΔG) consistently remained at ~-10 kcal mol^-1^ (**Fig 3E and 3F**), indicating the process is an energetically favourable spontaneous event. We have also performed additional control experiments showing that less than 5% of materials can be pelleted from solution after our ITC experiments using Gag and RNA (**S4A Fig**). Furthermore, similar amount of pelletable materials (<5%) were collected in parallel ITC experiments when nucleic acid free buffer was injected into the ITC chamber containing recombinant Gag (**S4B Fig**). These data implied that no virus-like-particles were generated in these ITC reactions under our experimental conditions when ‘low’ concentrations of Pr55^Gag^ and RNA were used (**S4 Fig**).

**Fig 5:**
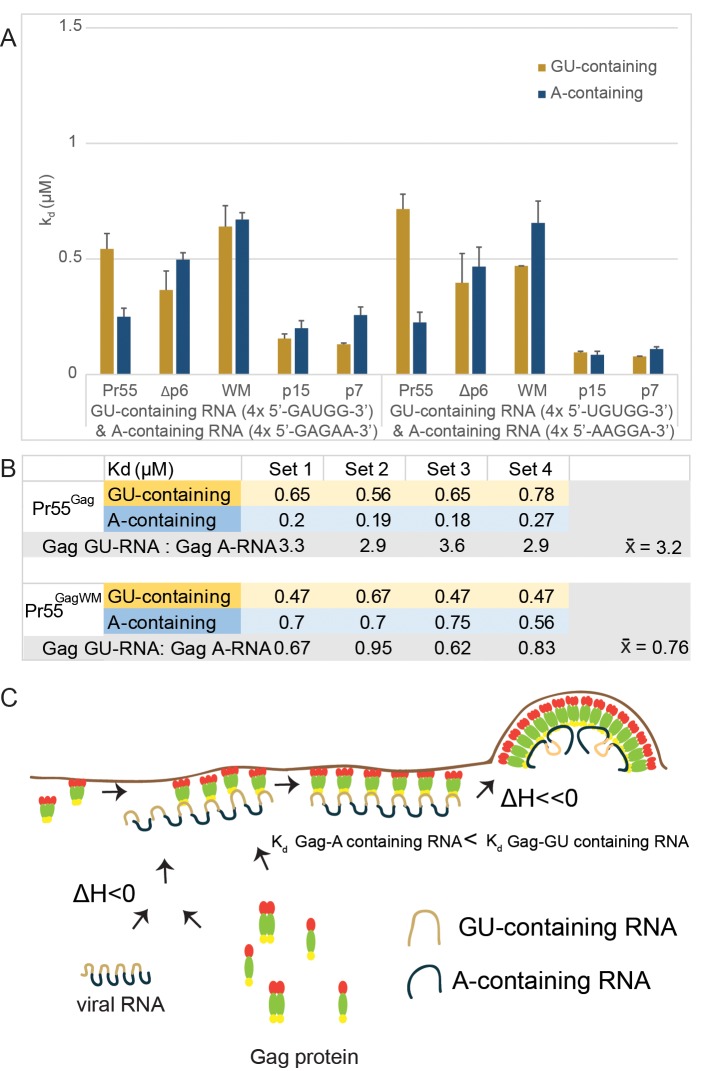
Oligomeric Gag has increased binding preference towards A-containing over GU-containing RNA sequences. **(A)** Calculated binding affinities (*K*_*d*_) from ITC binding curves fitted to a one-site interaction model are plotted for replicate interactions of first set of GU-containing and A-containing RNA (4x 5’-GUAGG-3’ and 4x 5’-GAGAA-3’, respectively) and second set of GU-containing and A-containing RNA (4x 5’-UGUGG-3’ and 4x 5’-AAGGA-3’, respectively) with Pr55^Gag^, Pr50^GagΔp6^, Pr55^GagWM^, p15^NC-SP2-p6^ and p7^NC^. **(B)** Calculated binding affinities (*K*_*d*_) for Pr55^Gag^ and Pr55^GagWM^ for replicate sets of interactions were tabulated and the fold difference between GU-containing and A-containing RNA binding affinities for each set were calculated. The average fold difference across the 4 sets is highlighted in bold. Sets 1 and 2 represent *K*_*d*_ values obtained from replicate Gag interactions with 4x 5’-GUAGG-3’ (GU-containing RNA) and 4x 5’-GAGAA-3’ (A-containing RNA). Sets 3 and 4 represent *K*_*d*_ values obtained from replicate Gag interactions with a second set of GU-containing and A-containing RNA (4x 5’-UGUGG-3’ and 4x 5’-AAGGA-3’, respectively). **(C)** Proposed model for Pr55^Gag^ trafficking and viral RNA interaction during viral assembly. The schematic representation of GU-containing and A-containing RNA motifs aims to represent how Gag interacts with different RNA motifs during virus biogenesis. The distributions of these RNA motifs are not evenly spread-out across HIV RNA genome, and more precise distributions of these RNA can be seen in **S3 Fig**.

In addition to the more favourable binding enthalpy of A-containing RNA to various Gag constructs, calculated binding affinities (*k*_*d*_) from ITC A-containing and GU-containing RNA binding curves revealed that oligomeric capable Pr55^Gag^ displayed ~3-times stronger binding affinity for the immature virus A-containing RNA motif over the cytosolic GU-containing RNA motif (**Fig 5A and 5B**). This occurrence was not observed when oligomerization-impaired forms of Gag (Pr55^Gag WM^) were used in parallel experiments (**Fig 5A and 5B**). Unexpectedly, Pr50^Δp6Gag^, which is capable of high-order Gag oligomerization, also did not display binding preference toward A-containing RNA motif (**Fig 5A and 5B**). It is possible that the p6 domain in Pr55^Gag^ plays a part in facilitating the binding of A-containing RNA to oligomeric forms of Pr55^Gag^, implying a previously unknown role of p6 late domain in the packing of Pr55^Gag^-Pr55^Gag^ and/or Pr55^Gag^-RNA interaction at the late stage of HIV assembly.

### Favourable interaction energetics between high-order Gag oligomers and Adenosine (A)-containing RNA persists with authentic HIV A-containing sequences

**Fig 6:**
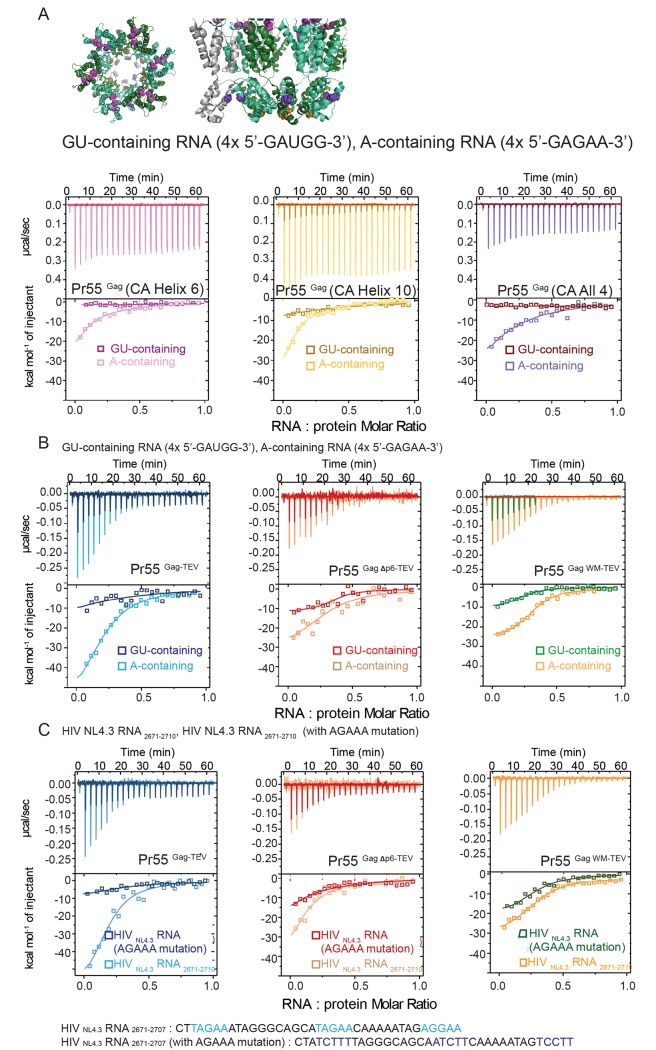
Favourable interaction energetics between high-order Pr55^Gag^ oligomers and Adenosine-containing RNA persists with authentic HIV A-containing sequences. **(A)** On the left is a top-down view of the published immature Pr55^Gag^ capsid hexamer cryo-EM structure (PDB ID:4usn) [32]. Intra-hexameric subunits are coloured in alternating green-cyan and dark green. Helix6 mutations (TTSTLQ 239–44 AASALA) disrupting intra-hexameric interactions of the NTD-capsid are shown as spheres and coloured in magenta. Helix10 mutation (D329A) and MHR mutation (K290A) disrupting intra-hexameric interactions of the CTD-capsid are shown as spheres and coloured in orange and blue-white, respectively. On the right is the side view of immature Pr55^Gag^ capsid. The WM mutation (WM316-7AA) disrupting inter-hexameric dimerization is highlighted in blue. Pr55^Gag^ (CA All 4) contains 4 sets of mutations that disrupt CA oligomerization (Helix6, Helix10, WM and MHR) [28]. ITC binding curves indicating heat released per mole of oligonucleotide titrated when 40 μM of 4x 5’-GAUGG-3’ (GU-containing) and 4x 5’-GAGAA-3’ (A-containing) RNA were injected in 1.5 ul aliquots into 8 μM of Pr55^Gag^ (CA Helix 6) (Fig 6A left panel, dark pink and light pink), Pr55^Gag^ (CA Helix 10) (Fig 6A middle panel, dark yellow and light yellow), and Pr55^Gag^ (CA All 4) (Fig 6A right panel, red-brown and purple). (n = 3) **(B)** Representative ITC binding curves indicating heat released per mole of oligonucleotide titrated when 40 μM of 4x 5’-GAUGG-3’ (GU-containing) and 4x 5’-GAGAA-3’ (A-containing) RNA were injected in 1.5 ul aliquots into 8 μM of Pr55^Gag-Tev^ (Fig 6B left panel, dark blue and light blue), Pr50^Gag Δp6-TEV^ (Fig 6B middle panel, dark orange and light orange), and Pr55^Gag WM-TEV^ (Fig 6B right panel, green and dark yellow). (n = 3) **(C)** Representative ITC binding curves indicating heat released per mole of oligonucleotide titrated when 40 μM of 5’- CTTAGAAATAGGGCAGCATAGAACAAAAATAGAGGAA-3’ (HIV_NL4.3_RNA_2671-2710_) and 5’- CTATCTTTTAGGGCAGCAATCTTCAAAAATAGTCCTT-3’ (HIV_NL4.3_RNA_2671-2710_ with AGAAA mutation) RNA were injected in 1.5 ul aliquots into 8 μM of Pr55^Gag-Tev^ (Fig 6C left panel, dark blue and light blue), Pr50^Gag Δp6-TEV^ (Fig 6C middle panel, dark orange and light orange), and Pr55^Gag WM-TEV^ (Fig 6C right panel, green and dark yellow). (n = 3)

To independently assess whether cytosolic low-order Gag oligomer binding with A-containing RNA is a less energetically favourable process than the interaction between high-order Gag oligomer and A-containing RNA, three additional Gag oligomerization defective mutants were engineered for analysis. These Gag oligomierization-impaired mutants are: (1) Pr55^Gag^ (CA Helix 6 mutations, TTSTLQ 239–44 AASALA), Pr55^Gag^ [CA Helix 6] (Fig 6A left panel) [28, 31]; (2) Pr55^Gag^ (CA Helix 10 mutation, D329A), Pr55^Gag^ [CA Helix 10] (Fig 6A middle panel) [28, 31]; and (3) multiple sites Gag oligomerization mutant (designated Pr55^Gag^ [CA All 4]) (Fig 6A right panel) that includes 4 sets of mutations at the: Pr55^Gag^ (CA dimerization interface WM 316–7 AA), Pr55^Gag^ (CA helix 6 TTSTLQ 239–44 AASALA), Pr55^Gag^ (CA helix 10 D329A), and Pr55^Gag^ CA major homology region (MHR K290A) [28]. These respective mutations (ie helix 6, helix 10 and MHR mutations) were chosen based on their reported inhibitory effects on viral assembly [28, 31], likely through their disruption of important intra-hexameric contacts within the immature CA hexamer [32] (**Fig 6A**). ITC analyses of these three Pr55^Gag^ oligomerization impaired mutants with the 20mer of cytosol GU-containing RNA (4x 5’-GAUGG-3’) showed minimal to no detectable binding (**Fig 6A**). In contrast, the binding of these same mutants with the 20mer of immature HIV A-containing RNA (4x 5’-GAGAA-3’) have led to energy release in the range of [ΔH ~ -18-24 kcal mol^-1^] (**Fig 6A**). These data are consistent with that of the previously described oligomerization impaired mutant, Pr55^Gag WM^ (**Fig 2**), showing that oligomerization of Pr55^Gag^ can help to increase the level of energy being released during Pr55^Gag^-RNA interaction in HIV assembly.

Given that the C-terminus His-Tag is present in all of the recombinant Gag used in this study thus far, it is unlikely that the His-Tag would selectively interfere with the binding for one of these three groups of RNA (Psi RNA, GU-containing RNA, and A-containing RNA) with Gag. However, to directly rule out any potential selective bias that might be introduced by the C-terminus His-Tag, a Tobacco Etch Virus (TEV) protease cleavage site was engineered in between the C-terminus end of Gag sequences and the His Tag for Pr55^Gag^, Pr50^GagΔp6^, and Pr55^Gag WM^. The His-Tag was removed via TEV protease digestion, and the His-Tag free recombinant Gag proteins (Pr55^Gag-TEV^, Pr50^GagΔp6-TEV^, and Pr55^Gag WM -TEV^) were subsequently column purified for analysis. ITC analyses of Pr55^Gag-TEV^ [**Fig 6B** left panel], Pr50^GagΔp6-TEV^ [**Fig 6B** middle panel], and Pr55^Gag WM-TEV^ [**Fig 6B** right panel] with either GU-containing RNA (4x 5’-GAUGG-3’) or A-containing RNA (4x 5’-GAGAA-3’) resulted in energy release ranging from ΔH ~ -8 kcal mol^-1^ to ~ -50kcal mol^-1^ (Fig 6B). More specifically, the ΔH are: (1) ~-10 kcal mol^-1^ for Pr55^Gag-TEV^-GU-containing RNA binding [**Fig 6B** left panel, dark blue]; (2) ~-10 kcal mol^-1^ for Pr50^Gag Δp6-TEV^-GU-containing RNA binding [**Fig 6B** middle panel, dark orange]; (3) ~-10 kcal mol^-1^ for Pr55^Gag WM-TEV^-GU-containing RNA binding [**Fig 6B** right panel, green]; (4) ~-45 kcal mol^-1^ for Pr55^Gag-TEV^-A-containing RNA binding [**Fig 6B** left panel, light blue]; (5) ~-25 kcal mol^-1^ for Pr50^Gag Δp6-TEV^-A-containing RNA binding [**Fig 6B** middle panel, light orange]; (3) ~-25 kcal mol^-1^ for Pr55^Gag WM-TEV^-A-containing RNA binding [**Fig 6B** right panel, dark yellow]. Analogous to the ITC results using the His-Tag containing Gag (Pr55^Gag^, Pr50^GagΔp6^, and Pr55^Gag WM^) (**Fig 2**), Gag binding with A-containing RNA (4x 5’-GAGAA-3’) were consistently shown to be a more energetically favourable reaction than parallel analyses that used GU-containing RNA (4x 5’-GAUGG-3’) as substrates (**Fig 6B**). Furthermore, the binding between Pr55^Gag-TEV^ and A-containing RNA was a more thermodynamically favourable reaction than that between Pr50^GagΔp6-TEV^ and A-containing RNA, highlighting the potential role of p6 in this process. Although the C-terminus His-Tag has no selective bias on the overall data interpretation, it is important to acknowledge that reduced levels of enthalpy (ΔH) were also detected with the Gag-RNA binding when the C-terminus His-Tag was removed from the recombinant Gag (**Fig 2** vs **Fig 6B**), implying that the C-terminus His-Tag has consistently increased the amounts of enthalpy release during Gag-RNA interaction.

It is conceivable that the synthetic 20mer RNA with the 4 consecutive A-containing RNA repeats (4x 5’-GAGAA-3’) does not truly represent the binding between HIV Gag and RNA sequences during viral assembly. To directly examine the relationship between authentic HIV RNA sequences and HIV Pr55^Gag^ protein during viral assembly, a 37mer RNA fragment representing the coding region of HIV reverse transcriptase (RNA positions 2671–2707) was used for ITC analyses. Using CLIP-sequencing, this RNA sequence (HIV_NL4.3_RNA_2671-2707_), consisting 3 independent A-containing RNA motifs, has been previously identified to be important for Pr55^Gag^ binding in immature HIV [21]. The same 37mer RNA fragment with mutations of the AGAAA RNA motifs (HIV_NL4.3_RNA_2671-2707_ with AGAAA mutation) was used as a control. ITC analyses of Pr55^Gag-TEV^, Pr50^GagΔp6-TEV^, and Pr55^Gag WM-TEV^ with the 37 mer RNA fragment HIV_NL4.3_RNA_2671-2707_ resulted in energy release ranging from ΔH of ~ -30 kcal mol^-1^ to ~ -45kcal mol^-1^ (**Fig 6C**), and the level of energy release with Pr55^Gag-TEV^ was 50% and 28% more than when Pr50^GagΔp6-TEV^ and Pr55^Gag WM-TEV^ were used, respectively (**Fig 6C**). In contrast, mutations of these identified A-containing RNA motifs within these authentic HIV RNA sequences eliminated most to all of its bindings with HIV Gag proteins (**Fig 6C**). The distinct level of energy release between Pr55^Gag-TEV^ and Pr50^GagΔp6-TEV^ based ITC experiments further support a potential role of p6 in the Gag-RNA and/or Gag-Gag interaction at the late stage of HIV assembly.

Overall, our ITC analyses provide direct evidence that the interaction between HIV Gag and RNA for Pr55^Gag^ oligomerization is an energetically (ΔG<0) favourable reaction, and it is associated with a favourable enthalpy (ΔH) and unfavourable entropy (ΔS). There is a general binding preference of HIV Gag toward A-containing RNA during viral assembly. Unexpectedly, our data have revealed that the p6 domain within Pr55^Gag^ also has a role in this Pr55^Gag^-A-containing RNA binding preference during virion biogenesis, which has raised a new question on a novel contribution of the p6 domain in the process of Gag-RNA interaction during viral assembly.

## Discussion

Our thermodynamic analyses showed that Pr55^Gag^-RNA binding was energetically favourable in terms of free energy exchange (ΔG<0) and that both Pr55^Gag^-Pr55^Gag^ interactions and the type of RNA sequence can contribute to the favourable change in enthalpy. Many studies have focused on the interaction of nucleic acids with the mature forms of the NC domain to probe the chaperone activity [16, 33] and nucleic acid binding properties of NC during reverse transcription [34, 35]. Our ITC studies represent the first thermodynamic characterization of the binding interaction between nucleic acids and full length Pr55^Gag^, allowing us to study the effects of Pr55^Gag^-Pr55^Gag^ interaction and Pr55^Gag^-RNA interaction on energy release. In the context of Pr55^Gag^ assembly, these ITC analyses reflect the net energy exchange when nucleic acids are injected into the Pr55^Gag^-Pr55^Gag^ and Pr55^Gag^-RNA complexes. It is important to emphasize that some of the observed energy exchange detected via ITC would be the consequence of Pr55^Gag^-Pr55^Gag^ oligomerization. Our analyses between Pr55^Gag^ and SL3 RNA suggested that at least 40% of energy release in our ITC experiment is derived from Pr55^Gag^ oligomerization. Our ITC study with RNA and full length Pr55^Gag^ (that is capable of forming higher-order Pr55^Gag^ oligomers) suggest that the additional release of energy from Pr55^Gag^-Pr55^Gag^ interactions could drive the binding of Pr55^Gag^ with nucleic acid and the oligomerization of Pr55^Gag^. This interpretation is consistent with recent *in vitro* membrane-bound Pr55^Gag^ assembly data showing that the genomic RNA selection by Pr55^Gag^ and the self-assembly of Pr55^Gag^ are interdependent [36].

Likewise, our observation that the Pr55^Gag^ binding with immature HIV A-containing RNA motifs has a greater ΔH than Pr55^Gag^ binding with the GU-containing cytosolic Pr55^Gag^-interacting RNA motifs during HIV biogenesis. These data suggest the additional heat release (that is associated with a greater entropic penalty) could help drive the Pr55^Gag^-complex to interact preferentially with A-containing RNA sequences during assembly. Furthermore, our thermodynamic analyses indicated that the oligomeric capable Pr55^Gag^ has 3-times greater affinity for immature virus A-containing RNA motifs compared to cytosolic GU-containing RNA sequences, but that oligomerization-impaired form of Gag (Pr55^Gag WM^) does not. Similar to Pr55^Gag WM^, other oligomerization-impaired forms of Gag (Pr55^Gag^ [CA Helix6], Pr55^Gag^ [CA Helix 10] and Pr55^Gag^ [CA All 4]) are consistent to have a less favorable [ΔH] in comparison to the wild type Pr55^Gag^ and A-containing RNA ITC analyses. The nucleic acid sequence dependence of the binding enthalpy is likely related to the process of HIV assembly and virion genesis. Early work by Campbell and Rein [37] has shown that recombinant Pr50^GagΔp6^ has distinct behavior with different nucleic acids during *in vitro* assembly. Recent work by Kutluay *et al* have reported that HIV Pr55^Gag^ would have a different preference towards distinct RNA sequence during virion genesis [21], although the precise mechanistic details of this relationship between HIV Pr55^Gag^ and specific RNA sequences during viral assembly will require further investigation.

These data allow us to propose a model for how viral assembly steps are regulated. The unfavorable entropic reaction with A-containing RNA motif and an increased binding stoichiometry between Gag and A-containing RNA sequences are also in agreement with a much tighter packing of Pr55^Gag^ molecules in the immature virus stage over low-order oligomeric Pr55^Gag^ in the cytoplasm. It is known that the HIV-RNA genome has a bias towards A-containing codons [38, 39]. While many of these A-rich sequences might be a consequence of G to A hyper mutation from APOBEC3G pressure [40, 41], HIV has found ways to utilise them to its own gain. Indeed, these A-rich viral sequences are already known to be important for RNA trafficking from nucleus to cytoplasm via the RRE-Rev relationship [42, 43], and also for supporting the synthesis of viral cDNA during reverse transcription [44]. Now, we have evidence to suggest the A-rich RNA codon (in the form of identified A-containing RNA motifs) [21] may also have a role in regulating the viral assembly process thermodynamically. It is important to note that HIV Pr55^Gag^ consistently displayed more favourable enthalpy [ΔH] when it binds to A-containing RNA motifs over GU-containing RNA motifs, and it is remains to be determined why HIV Pr55^Gag^ has a binding preference with GU-containing RNA motifs in the cytoplasm and the plasma membrane [21]. One potential explanation could be that the context of how these GU-containing RNA motifs are being presented in HIV genomes (or cellular mRNA) is also an important determinant for Pr55^Gag^-RNA binding during HIV assembly, and further investigations are needed to dissect this process. Moreover, it would be important to highlight that the preference of Pr55^Gag^ toward A-containing RNA is in part associated with the p6 domain. This unexpected observation suggests a previous unknown role of p6 in Pr55^Gag^-RNA interaction, and how p6 (and potentially in conjunction with ESCRT protein) might achieve this mechanistically would require further evaluation.

Taken together, our findings show how the virus might derive the energy required to drive the Pr55^Gag^-Pr55^Gag^ assembly and the mechanism by which HIV-1 assembles. We propose a model (**Fig 5C**) wherein many Pr55^Gag^ molecules bind to GU-containing RNA in the cytosol forming low-order oligomers. The Pr55^Gag^-RNA complex then traffics to the plasma membrane and acts as a nucleation site for Pr55^Gag^ assembly; with energy released from Pr55^Gag^-Pr55^Gag^ and Pr55^Gag^-RNA interactions driving the formation of higher order Pr55^Gag^ oligomerization complexes. The high-order Pr55^Gag^ complex in turn interacts preferentially with A-containing viral RNA sequences with a lower *K*_*d*_, and the process is further enhanced by the additional release of heat through interaction with the A-containing viral RNA sequences to assist in the packaging of the genome, thus driving the completion of the particle formation.

## Materials and methods

### Production and purification

Recombinant Gag proteins (Pr55^Gag^, Pr50^Δp6Gag^, Pr55^Gag WM 316–7 AA^, Pr55^Gag^ [CA Helix 6 TTSTLQ 239–44 AASALA], Pr55^Gag^ [CA Helix 10 D329A], Pr55^Gag^ [CA All 4]) and processed NC proteins (p15^NC-SP2-p6^, p7^NC^) were expressed with C-term His-tag and purified as previously described [45]. Large scale production and purification of Pr55^Gag^ is described herein.

### Media

Defined medium (DM1) used for seed cultures contained per litre: KH_2_PO_4_, 13.3 g; (NH_4_)_2_HPO_4_, 4.0 g; citric acid, 1.7 g; glucose, 10 g; MgSO_4_.7H_2_O, 0.62 g; kanamycin, 50 mg; thiamine hydrochloride, 4.4 mg; and trace salts solution, 5 mL. Defined medium (DM2) used in the bioreactors contained per litre: KH_2_PO_4_, 10.6 g; (NH_4_)_2_HPO_4_, 4.0 g; citric acid, 1.7 g; glucose, 25 g; MgSO_4_.7H_2_O, 1.23 g; kanamycin, 50 mg; thiamine hydrochloride, 4.4 mg; and trace salts solution, 5 mL. The trace salts solution contained per litre: CuSO_4_.5H_2_O, 2.0 g; NaCI, 0.08 g; MnSO_4_.H_2_O, 3.0 g; Na_2_MoO_4_.2H_2_O, 0.2 g; boric acid, 0.02 g; CoCl_2_.6H_2_O, 0.5 g; ZnCl_2_, 7.0 g; FeSO_4_.7H_2_O, 22.0 g; CaSO_4_.2H_2_O, 0.5 g and H_2_SO_4_, 1 mL. As required, glucose, magnesium, trace salts, thiamine and kanamycin were aseptically added as concentrated stock solutions to media after sterilisation.

### Seed cultures

Primary seed cultures were prepared from single colonies taken from a fresh transformation plate, and grown in 10 mL of DM1 (in a 30 mL bottle). The cultures were incubated at 37°C shaking at 200 rpm for 23 h. A volume (0.5 mL) of the primary seed culture was used to inoculate 500mL of DM1 (in a 2 L Erlenmeyer flask). These secondary seed cultures were incubated at 37°C shaking at 200 rpm for 16 h.

### Protein expression in 2L stirred-tank bioreactors

Recombinant HIV Gag proteins were produced in 2 L stirred tank bioreactors connected to a Biostat B (Sartorius Stedim, Germany) control system. The initial volume of medium in the bioreactor was 1.6 L and glucose as used as the carbon source. A volume of the secondary seed culture was added to the bioreactor to attain an initial optical density (measured at 600 nm) of 0.25. Foaming was controlled via the automatic addition of 10% (v/v) polypropylene glycol 2025; 3 mL of the antifoam solution (Sigma, Antifoam 204) was added prior to inoculation. The pH set-point was 7.0 and controlled by automatic addition of either 10% (v/v) H_3_PO_4_ or 10% (v/v) NH_3_ solution. The dissolved oxygen set-point was 30% of saturation and a two-step cascade control was used to maintain the dissolved oxygen above the specified set-point. The agitator speed ranged from 500 rpm to 1200 rpm and airflow (supplemented with 5% pure O_2_) ranged from 0.3 L min^-1^ to 1.5 L min^-1^. The ratio of air to oxygen was manually changed as required. To assist with correct folding of the Gag proteins, the medium was supplemented with 50 μM ZnSO_4_ added 1.8 h after inoculation. A high cell density fed-batch process was used, with the feed solution comprised of 400 mL of 660 g L^-1^ glucose solution to which 40 mL of 1 M MgSO_4_.7H_2_O was added. The feed flow rate was 21 mL hr^-1^ and commenced once the initial glucose supply was exhausted (typically 8 to 9 hr after inoculation). Two hours after the fed-batch process was initiated the bioreactor temperature set-point was reduced to 18°C, with culture temperature dropping to 19°C within 30 min. After cooling, protein expression was induced via the addition of 1 mM isopropyl-β-D-thiogalactopyranoside (IPTG) and 0.02% (w/v) arabinose and the feed flow rate reduced to 4 mL hr^-1^. Cells were harvested by centrifugation (12000 *g*, 4°C, 10 min) 22 to 23 h after inoculation and cell pellets stored at -80°C.

### Protein purification

Cell pellets (~200 g) were thawed and re-suspended in 1.0 L of ice cold lysis buffer (1 M NaCl, 50 mM TRIS-HCl pH 8.0, 5 mM MgCl_2_, 10 mM imidazole, 1 (v/v)% Tween-20, 10% (v/v) glycerol, 5 mM DTT) containing 50,000 units of DNase I, 5 mM benzamidine-HCl and 1 mM phenyl methyl sulfonyl fluoride (PMSF). The cell suspension was homogenized (EmulsiFlex-C5 homogenizer, Avestin) pre-chilled to 4°C, three times at 700 bar pressure. The lysate was clarified by centrifugation 12,000 *g*, 4°C, 30 min), and the supernatant filtered using a 0.45 μm Stericup filter (Millipore).

### IMAC (Immobilized Metal Affinity Chromatography)

The supernatant was loaded at 5 mL min^-1^ onto a 30 mL HisTRAP fast flow IMAC column (consisting of 6 x 5 mL cartridges connected in series; GE Healthcare) using a MINIPULS peristaltic pump (Gilson). The column had previously been equilibrated with 5 column volumes (CV) binding buffer (1.0 M NaCl, 50 mM TRIS-HCl pH 8.0, 5 mM MgCl_2_, 10 mM imidazole, 1% (v/v) Tween-20, 10% (v/v) glycerol, 5 mM DTT). The column was washed with 10 CV wash buffer (1.0 M NaCl, 50 mM TRIS-HCl pH 8.0, 5 mM MgCl_2_, 25 mM imidazole, 1% (v/v) Tween-20, 10% (v/v) glycerol, 5 mM DTT) and bound proteins eluted with 5 CV elution buffer (1.0 M NaCl, 50 mM TRIS-HCl pH 8.0, 5 mM MgCl_2_, 250 mM imidazole, 1% (v/v) Tween-20, 10% (v/v) glycerol, 5mM DTT). Pr55^Gag^ eluting from the IMAC column was concentrated to ~ 3.0 mg mL^-1^ using a centrifugal concentrator (Amicon Ultra-15, 10,000 molecular weight cut-off membrane; Millipore).

### Size exclusion chromatography

The concentrated protein (5x 10 mL) was fractionated by size exclusion chromatography (SEC) using a Superdex 200 26/60 column (GE Healthcare) previously equilibrated in SEC buffer (50 mM TRIS-HCl, 1.0 M NaCl, 5 mM DTT, pH 8.0) using an ÄKTApurifier chromatography workstation (GE Healthcare). Peak fractions (UV 280nm) containing Pr55^Gag^ were collected, pooled and concentrated to 1–2 mg mL^-1^ as described above, and snap frozen in liquid nitrogen before storage at -80°C.

### TEV cleavage

Purified protein containing the TEV sequence is digested with 1:25 (w/w) TEV protease (produced in-house) at 4°C for 14 hrs. Efficiency of the cleavage is assessed by sodium dodecyl sulfate—poly acrylamide gel electrophoresis (SDS-PAGE). A 1ml NI-NTA column pre-equilibrated with SEC buffer and the TEV digested protein is applied to the column and the flowthrough is collected and passed over the column two more times. The cleaved His6-Tag and uncut fusion protein is eluted from the column using 5 CV elution buffer (1.0 M NaCl, 50 mM TRIS-HCl pH 8.0, 5 mM MgCl_2_, 250 mM imidazole, 1% (v/v) Tween-20, 10% (v/v) glycerol, 5mM DTT).

### Crosslinking assay

Protein was buffer exchanged into 1x Na/K 10mM phosphate buffer (pH 7.4) with different NaCl concentrations and concentrated to 1 mg mL^-1^. Yeast tRNA (Sigma Aldrich) (10% (w/w) ratio of nucleic acid to protein) was added to the protein solution and incubated for 30 min at room temperature, followed by addition of paraformaldehyde (PFA) (final concentration of 0.2% w/v). After incubating the solution for further 30 min at room temperature, the crosslinking was stopped by addition of 50 μL of 3M TRIS pH 8.0. Samples were centrifuged 10 min at 10,000 *g* and supernatants were analyzed by size exclusion chromatography using a Superdex 200 10/30 column (GE Healthcare), previously equilibrated in phosphate-buffered saline (PBS) with respective NaCl concentrations. Peak fractions were concentrated to 2 mg mL^-1^ as previously described. Fractions were electrophorised on a NuPAGE Novex 3–8% TRIS-Acetate protein gel under denaturing conditions before being transferred onto nitrocellulose membranes for Western analysis.

### Electron microscopy

Pr55^Gag^ and Pr55^GagΔp6^ were concentrated to 2.0 mg mL^-1^ in 50 mM TRIS pH 8.0 containing 1.0 M NaCl and 10 mM dithiothreitol and mixed with TG30 at a nucleic acid to protein ration of 4% (w/w) prior to dialysis against 50 mM TRIS pH 8.0 containing 150 mM NaCl and 10 mM dithiothreitol overnight at 4°C. Particles were imaged using both negative stain transmission electron microscopy and cryo-electron microscopy.

For negative stain imaging, the stock solution was diluted 100-fold to give a single layer of well-separated particles in most fields of view. Carbon-coated grids were glow discharged in nitrogen prior to use to facilitate sample spreading. Aliquots of approximately 4 μL were pipetted onto each grid and allowed to settle for 30 s. Excess sample was drawn off with filter paper, and the remaining sample stained with a drop of 2% aqueous phosphotungstic acid. Again, excess liquid was drawn off with filter paper. Grids were air dried until required. Samples were examined using a Tecnai 12 Transmission Electron Microscope (FEI, Eindhoven) at an operating voltage of 120kV. Images were recorded using a Megaview III CCD camera and AnalySIS camera control software (Olympus.)

For cryo-electron microscopy, virus particles were prepared, processed and imaged as previously described [46].

### Sucrose gradient

For the nucleic acid mediated *in vitro* assembly, protein (2.5 mg mL^-1^) and a DNA 30-mer oligonucleotide with alternating TG motifs (TG30; Macrogen) at a 10% (w/w) ratio of nucleic acid to protein were mixed in 50 mM TRIS, 500 mM NaCl, pH8.0 prior to adding inositol hexaphosphate (IP6) (10 μM) and slowly decreasing the NaCl concentration by dialysis into 50 mM TRIS, 150 mM NaCl, pH8.0 buffer to initiate the assembly process. The buoyant density of the particles produced and their *in vitro* assembly efficiency was assessed by layering the assembly reaction mixture onto a 32.5 to 55% (w/w) linear sucrose gradient in TBS (150 mM NaCl, 50 mM Tris, pH 7.6). Assembled HIV-1 Gag samples were layered onto the gradient and centrifuged for 16 h at 110,000 *g* (SW41 rotor: Optima L-90k ultracentrifuge; Beckman). 750 μL fractions were collected from the top in separate 1.5 mL tubes after completion of the run and prepared for trichloroacetic acid (TCA) precipitation.

### TCA precipitation

350 μL of 50% (w/v) TCA was added to each fraction and incubated at 4°C for 30 min. Tubes were centrifuged at 14,000 *g* for 10 min at 4°C and the pellet was resuspended in 200 μL of prechilled acetone and incubated for 5 min at 4°C. The centrifugation step was repeated and excess supernatant aspirated and tubes air dried. The dried pellet was resuspended in 20 μL of 4x NuPAGE reduced LDS Sample Buffer (Life Technologies) and heated for 5 min at 95°C. Samples were briefly centrifuged and 10 μL of each sample was loaded onto a NuPAGE 4–12% Bis-TRIS Midi gel and electrophoresed using NuPAGE MES SDS Running Buffer (Life Technologies) at 150 volts.

### Western blot

PAGE gels were transferred to a Nitrocellulose membrane (PerkinElmer) using XCell II Blot Module (30 volts for 60 mins), and the membrane blocked overnight in blotto (5% (w/v) skim milk powder in TBS + 0.05% (v/v) Tween-20) at 4°C. Membrane was washed 3x with wash buffer (TBS + 0.05% (v/v) Tween-20) for 5 min each and probed with a mouse anti-CA monoclonal antibody (Hybridoma Clone 183-H12-5C; NIH AIDS Reagent program) for 1 h at room temperature. Membrane was washed 3x with wash buffer, incubated with a secondary goat anti-mouse IRDye 800CW conjugate (LI-COR; diluted1:30,000 in blotto) for 1 hr at room temperature. Membrane was washed again 3x and analysed using an ODYSSEY CLx system (LI-COR).

### Isothermal titration calorimetry

Isothermal titration calorimetry (ITC) experiments were performed at 30°C using a Microcal Auto-ITC_200_ MicroCalorimeter (Malvern). For each ITC experiment, the cell contained soluble Gag protein (6–10 μM) in TBS with 1mM tris (2-carboxyethyl) phosphine (TCEP) and the syringe contained 15–50 μM of the DNA 30-mer oligonucleotide with alternating TG motifs (TG30) or RNA 20-mer oligonucleotides with 4 consecutive repeating GU-containing (4x 5’-GAUGG-3’; 4x 5’-UGUGG-3’) or A-containing (4x 5’-GAGAA-3’; 4x 5’-AAGGA-3’) sequence motifs. The SL3 DNA and the 20mer SL3 RNA (5’GGACUAGCGGAGGCUAGUCC3’) as well as the HIV_NL4.3_RNA_2671-2707_ (5’-CTTAGAAATAGGGCAGCATAGAACAAAAATAGAGGAA-3’) and the HIV_NL4.3_RNA_2671-2707_ (with AGAAA mutation) (5’-CTATCTTTTAGGGCAGCAATCTTCAAAAATAGTCCTT-3’) are based on NL4.3 HIV proviral RNA. DNA and RNA oligonucleotides were purchased from Macrogen and Integrated DNA Technologies, respectively, and dissolved in TBS with 1mM TCEP. The volume of the first injection for each ITC run was set to 0.4 μL over 0.8 s to minimize the experimental impact caused by dilution effects at the injection syringe tip; this initial injection was excluded from data analysis. The first injection was followed by 25 injections of 1.5 μL over 3 s or 38 injections of 1 μL over 2 s each with the interval between each injection set to 300 s. The reference power was set to 5 μcals^-1^. The syringe stirring speed was set to 750 rpm.

A baseline was drawn by linear extrapolation using the data points collected from control experiments and subtracted from the whole data set to correct for the heat of dilution. The total heat signal from each injection was determined as the area under the individual peaks and plotted against the [nucleic acid]/[Gag] molar ratio. The corrected data were analyzed to determine number of binding sites (*n*), and molar change in enthalpy of binding (ΔH) in terms of a single site model derived as follows:

The quantity r is defined as the moles of nucleic acid [D] bound per mole of protein [P] with an association constant (*K*_*a*_):

$$r=\frac{K_{a}[D]}{1+K_{a}[D]}$$ (Eq 1)

Solving *Eq* 1 for K_a_ leads to:

$$K_{a}=\frac{r}{\left(1+r)[D\right]}$$ (Eq 2)

Since the total concentration of nucleic acid [D]_T_ in the cell is known, it can be represented by *Eq* 3 wherein [P]_T_ equals the total protein concentration in the cell and *n* the number of binding sites:

$${\left[D\right]}_{T}=[D]+nr{\left[P\right]}_{T}$$ (Eq 3)

Since *Eq* 3 shows *nr*[P]_T_ = [PD], the fraction of sites occupied by the nucleic acid, combining *Eqs* 2 and 3 leads to:

$$r^{2}-r\left[\frac{{\left[D\right]}_{T}}{n{\left[P\right]}_{T}}+\frac{1}{nK_{a}{\left[P\right]}_{T}}+1\right]+\frac{{\left[D\right]}_{T}}{n{\left[P\right]}_{T}}=0$$ (Eq 4)

Solving the quadratic for the fractional occupancy (*r*) gives:

$$r=\frac{1}{2}\left[(\frac{{\left[D\right]}_{T}}{n{\left[P\right]}_{T}}+\frac{1}{nK_{a}{\left[P\right]}_{T}}+1)-\sqrt{{\left(\frac{{\left[D\right]}_{T}}{n{\left[P\right]}_{T}}+\frac{1}{n{{K_{a}[P]}_{T}}_{}}+1\right)}^{2}-\frac{4{\left[D\right]}_{T}}{n{\left[P\right]}_{T}}}\right]$$ (Eq 5)

The total heat content (Q) of the solution in the volume of the sample cell (V_o_; determined relative to zero for the *apo*-species) at fractional saturation *r* is given by *Eq* 6, where ΔH represents the molar heat of nucleic acid binding:

$$Q=nr{\left[P\right]}_{T}\Delta HV_{0}$$ (Eq 6)

Substituting *Eq* 5 into *Eq* 6 gives:

$$Q=\frac{nr{\left[P\right]}_{T}\Delta HV_{0}}{2}\left[(\frac{{\left[D\right]}_{T}}{n{\left[P\right]}_{T}}+\frac{1}{nK_{a}{\left[P\right]}_{T}}+1)-\sqrt{{\left(\frac{{\left[D\right]}_{T}}{n{\left[P\right]}_{T}}+\frac{1}{nK_{a}{\left[P\right]}_{T}}+1\right)}^{2}-\frac{4{\left[D\right]}_{T}}{n{\left[P\right]}_{T}}}\right]$$ (Eq 7)

The total heat content, Q can be calculated as function of *n*, *K*_*a*_, ΔH because [P]_T_, [D]_T_ and V_o_ are known experimental parameters. The parameter Q defined in *Eq* 7 only applies to the known starting volume of protein solution in the sample cell (V_o_). In order to correct for the displaced volume (V_i_), the change in heat content Q(i) at the end of the i^th^ injection is defined by *Eq* 8 to obtain the best fit for *n*, *K*_*a*_, and ΔH by standard Marquardt methods until no further significant improvement in fit occurs with continued iteration.

$$\Delta Q(i)=Q(i)+\frac{dVi}{Vo}\left[\frac{Q(i)+Q(i-1)}{2}\right]-Q(i-1)$$ (Eq 8)

The Gibbs free energy (ΔG^0^) was calculated from the fundamental equation of thermodynamics *Eq* 9:

ΔG°=ΔH−TΔS=−RTlnKa (Eq 9)

All data fitting operations were performed with Origin V7.0 software (OriginLab, Northampton, MA).

### Protein estimation post-ITC analysis

Following ITC analysis, the solutions containing HIV protein and RNA complex was spun at *100*,*000 g* for 1 hr (TLA 100.2 rotor; optima max ultracentrifuge; Beckman). The supernatant was removed and the pellet was resuspended in 50μl of TBS. Protein estimation was done using UV-Vis (A280) on both the supernatant and the pelletable materials (NanoDrop1000; Thermo Scientific) via Bradford Protein Assay [47].

## Supporting information

S1 Fig **Increase in production of recombinant His-tagged HIV-1 Pr55**^**Gag**^ Cryo EM analysis of mammalian cell derived VLP of **(A)** Wild type Gag and **(B)** C-terminus His-tagged Gag. **(C)** Gel filtration profile of the purified recombinant Pr55^Gag^ with the bioreactor protocol, compared to the previously published purification method. Peak fractions were analysed with **(D)** SDS-PAGE and **(E)** Western blot using an anti-capsid antibody.
(TIF)

S2 Fig Pr55^Gag^ and Pr50^GagΔp6^ follow similar oligomerization and assembly pattern. *In vitro* assembled particles from recombinant **(A)** Pr55^Gag^ and **(B)** Pr50^GagΔp6^ were negatively stained and analysed using Transmission Electron Microscopy (TEM). Size exclusion chromatography (SEC) of **(C)** Pr55^Gag^ and **(D)** Pr50^GagΔp6^ assembled in the presence of nucleic acid and crosslinked. Blue trace represents gel filtration profile in the absence of nucleic acid. Fractions following SEC were analysed by Western blotting using anti-CA for both **(E)** Pr55^Gag^ and **(F)** Pr50^GagΔp6^. Sucrose gradient analysis of mammalian cell derived VLPs from cells transfected with **(G)** wild type Gag and C-Terminus His Tagged Gag. Analysis indicates VLPs derived from mammalian cells are mostly observed in fractions 6–10 (boxed). Sucrose gradients showed that adding IP6 to the assembly of recombinant **(H)** Pr55^Gag^ and **(I)** Pr50^GagΔp6^ promoted formation of higher order complexes, which exhibited similar fractionation positions as with mammalian cell derived Gag VLP.
(TIF)

S3 Fig GU-containing and A-containing RNA motifs interspersed within the HIV genome. Schematic of HIV-1 genome with **(A)** approximate sequence positions of GU-containing motifs [4x 5’-GAUGG-3’ (red) and 4x 5’-UGUGG-3’ (dark red)] and **(B)** approximate sequence positions A-containing motifs [4x 5’-GAGAA-3’(blue) and 4x 5’-AAGGA-3’(dark blue)] within the complete HIV genome (NCBI Reference Sequence: NC_001802.1).
(TIF)

S4 Fig Virus-like-particles do not form during ITC reactions when ‘low’ concentration of proteins and RNA were used. Using spectroscopic analytical procedure as well as UV-Vis Spectrophotometry, the pelletable materials is expressed as percentage of the total initial input protein were estimated post-ITC reactions. Following ITC analyses using **(A)** A-containing (4x 5’-GAGAA-3’) RNA showing that >90% of the initial input protein still remained soluble. Control ITC experiment where RNA free buffer was injected into ITC chambers was also carried out simultaneously **(B)** as a parallel comparison. n = 3
(TIF)
