# Supplementary figures and images for: The thermodynamics of Pr55Gag-RNA interaction regulate the assembly of HIV
Source: PLoS Pathog. 2017 Feb 21;13(2):e1006221. doi: 10.1371/journal.ppat.1006221 (PMC5336307; doi:10.1371/journal.ppat.1006221)

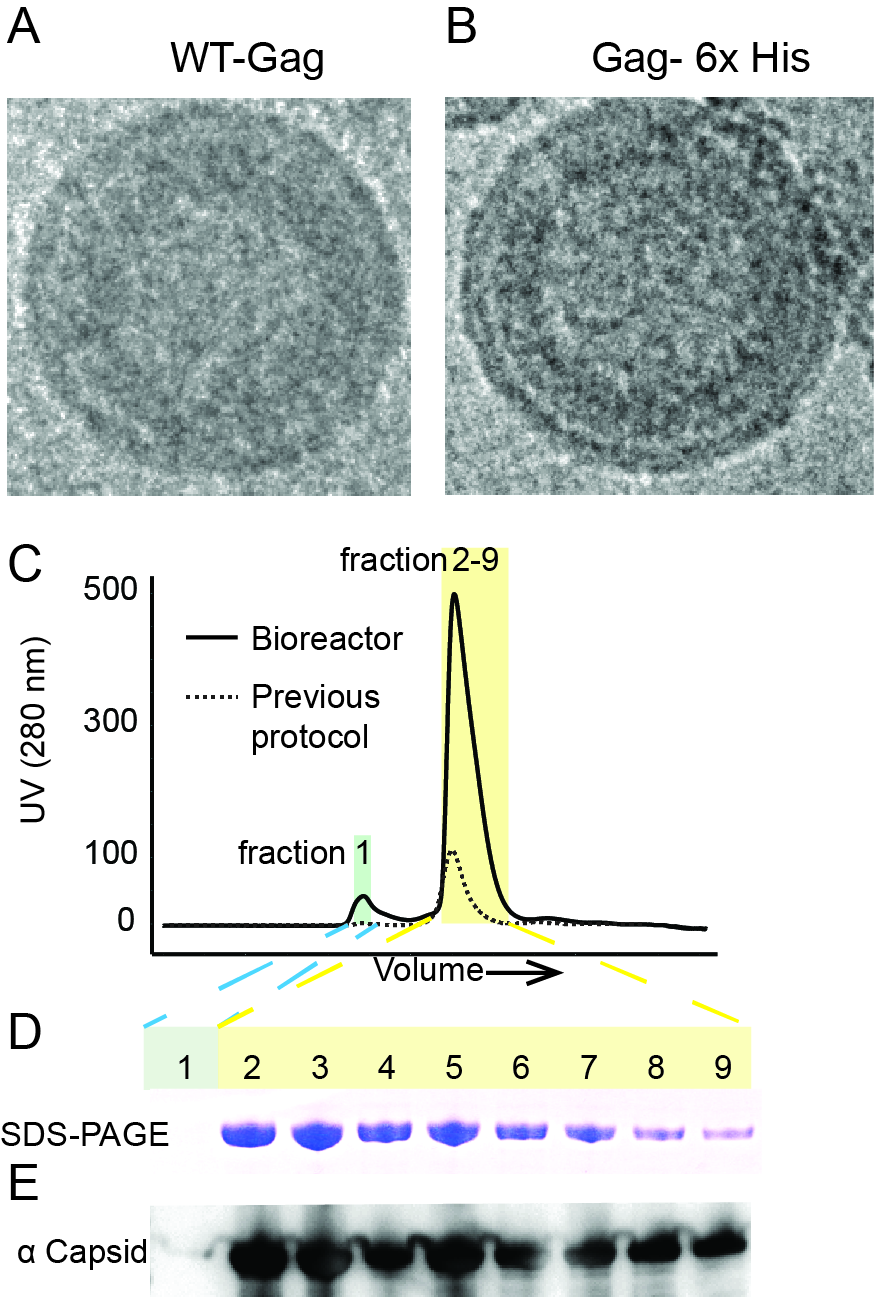

Supplement: S1 Fig — Increase in production of recombinant His-tagged HIV-1 Pr55Gag Cryo EM analysis of mammalian cell derived VLP of (A) Wild type Gag and (B) C-terminus His-tagged Gag. (C) Gel filtration profile of the purified recombinant Pr55Gag with the bioreactor protocol, compared to the previously published purification method. Peak fractions were analysed with (D) SDS-PAGE and (E) Western blot using an anti-capsid antibody. (TIF) [file ppat.1006221.s001.tif]

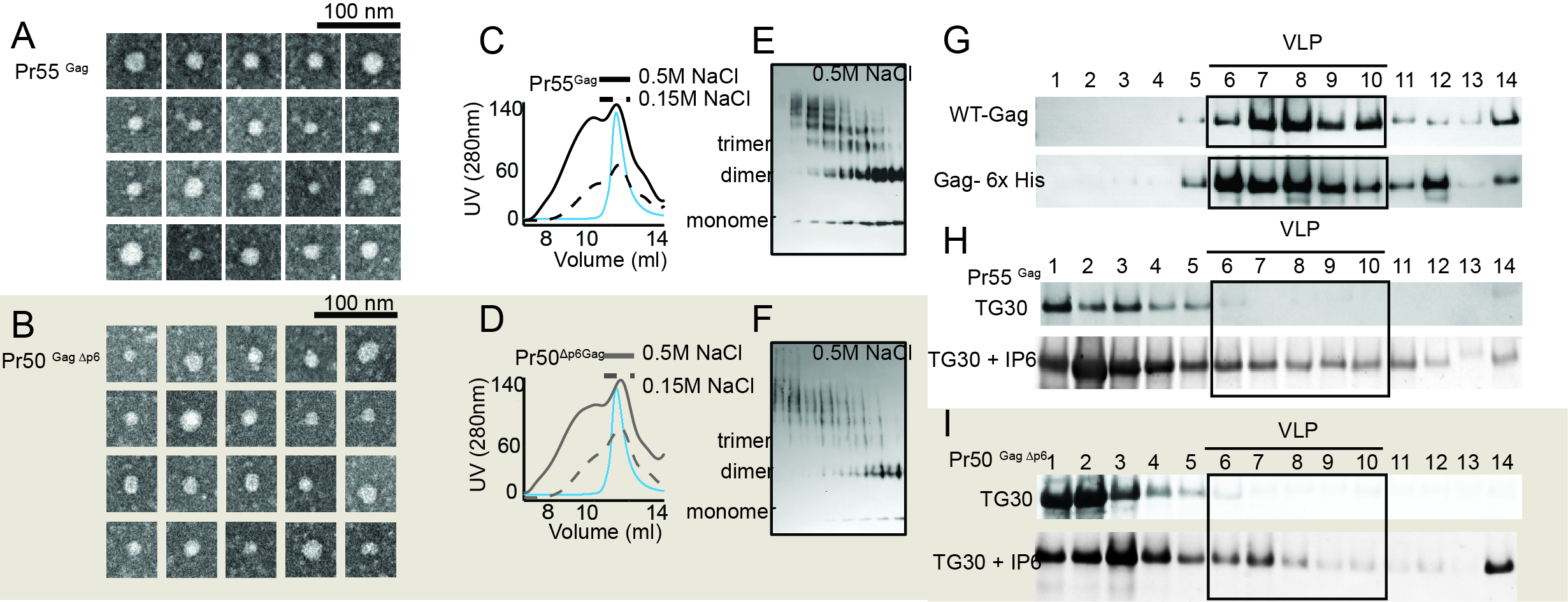

Supplement: S2 Fig — In vitro assembled particles from recombinant (A) Pr55Gag and (B) Pr50GagΔp6 were negatively stained and analysed using Transmission Electron Microscopy (TEM). Size exclusion chromatography (SEC) of (C) Pr55Gag and (D) Pr50GagΔp6 assembled in the presence of nucleic acid and crosslinked. Blue trace represents gel filtration profile in the absence of nucleic acid. Fractions following SEC were analysed by Western blotting using anti-CA for both (E) Pr55Gag and (F) Pr50GagΔp6. Sucrose gradient analysis of mammalian cell derived VLPs from cells transfected with (G) wild type Gag and C-Terminus His Tagged Gag. Analysis indicates VLPs derived from mammalian cells are mostly observed in fractions 6–10 (boxed). Sucrose gradients showed that adding IP6 to the assembly of recombinant (H) Pr55Gag and (I) Pr50GagΔp6 promoted formation of higher order complexes, which exhibited similar fractionation positions as with mammalian cell derived Gag VLP. (TIF) [file ppat.1006221.s002.tif]

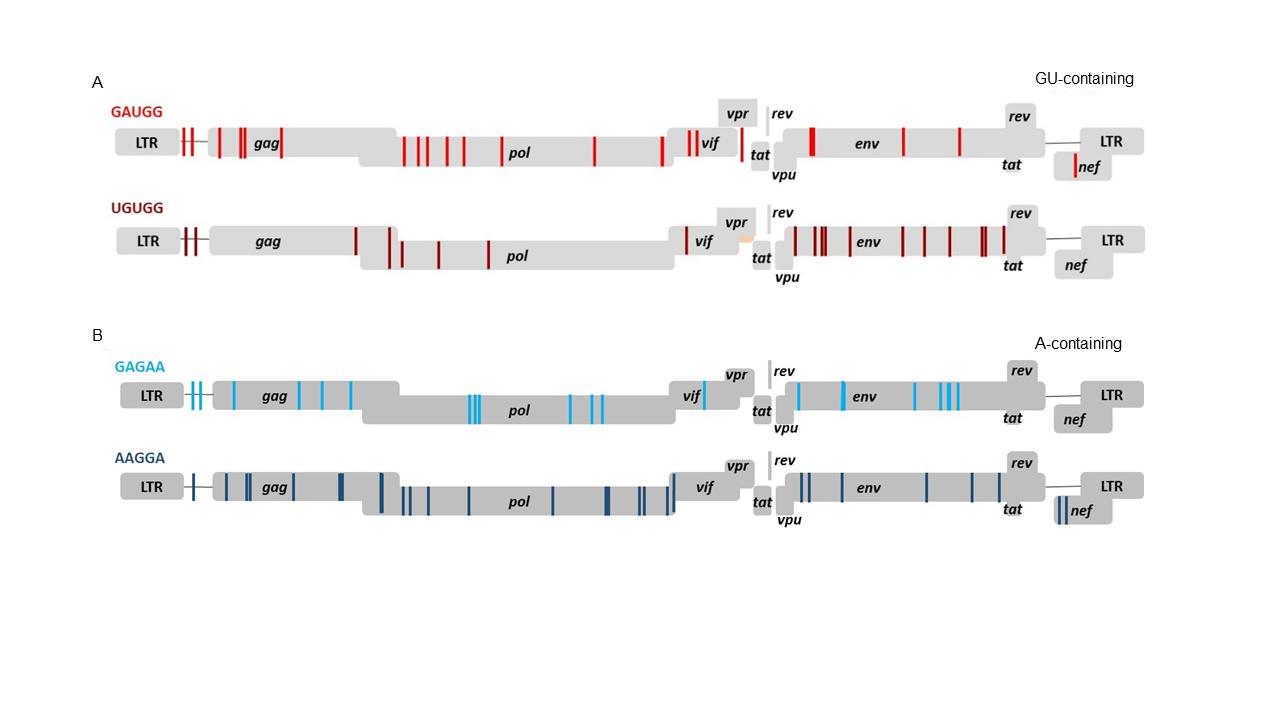

Supplement: S3 Fig — Schematic of HIV-1 genome with (A) approximate sequence positions of GU-containing motifs [4x 5’-GAUGG-3’ (red) and 4x 5’-UGUGG-3’ (dark red)] and (B) approximate sequence positions A-containing motifs [4x 5’-GAGAA-3’(blue) and 4x 5’-AAGGA-3’(dark blue)] within the complete HIV genome (NCBI Reference Sequence: NC_001802.1). (TIF) [file ppat.1006221.s003.tif]

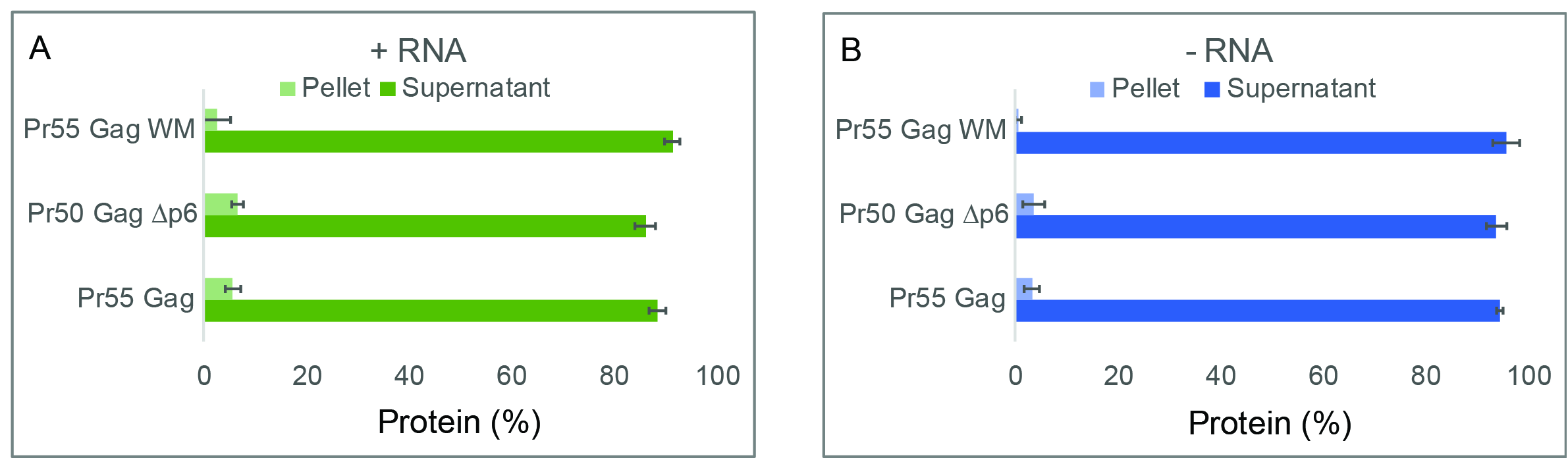

Supplement: S4 Fig — Using spectroscopic analytical procedure as well as UV-Vis Spectrophotometry, the pelletable materials is expressed as percentage of the total initial input protein were estimated post-ITC reactions. Following ITC analyses using (A) A-containing (4x 5’-GAGAA-3’) RNA showing that >90% of the initial input protein still remained soluble. Control ITC experiment where RNA free buffer was injected into ITC chambers was also carried out simultaneously (B) as a parallel comparison. n = 3 (TIF) [file ppat.1006221.s004.tif]
